# Structural, Culinary, Nutritional and Anti-Nutritional Properties of High Protein, Gluten Free, 100% Legume Pasta

**DOI:** 10.1371/journal.pone.0160721

**Published:** 2016-09-07

**Authors:** Karima Laleg, Denis Cassan, Cécile Barron, Pichan Prabhasankar, Valérie Micard

**Affiliations:** 1 UMR IATE, CIRAD, INRA, Montpellier SupAgro, Montpellier University, Montpellier, France; 2 CRNH Auvergne, UNH, UMR 1019, INRA, Clermont-Ferrand, France; 3 CSIR-Central Food Technological Research Institute, Mysore, India; Istituto di Biologia e Biotecnologia Agraria Consiglio Nazionale delle Ricerche, ITALY

## Abstract

Wheat pasta has a compact structure built by a gluten network entrapping starch granules resulting in a low glycemic index, but is nevertheless unsuitable for gluten-intolerant people. High protein gluten-free legume flours, rich in fibers, resistant starch and minerals are thus a good alternative for gluten-free pasta production. In this study, gluten-free pasta was produced exclusively from faba, lentil or black-gram flours. The relationship between their structure, their cooking and Rheological properties and their *in-vitro* starch digestion was analyzed and compared to cereal gluten-free commercial pasta. Trypsin inhibitory activity, phytic acid and α-galactosides were determined in flours and in cooked pasta. All legume pasta were rich in protein, resistant starch and fibers. They had a thick but weak protein network, which is built during the pasta cooking step. This particular structure altered pasta springiness and increased cooking losses. Black-gram pasta, which is especially rich in soluble fibers, differed from faba and lentil pasta, with high springiness (0.85 vs. 0.75) and less loss during cooking. In comparison to a commercial cereal gluten-free pasta, all the legume pasta lost less material during cooking but was less cohesive and springy. Interestingly, due to their particular composition and structure, lentil and faba pasta released their starch more slowly than the commercial gluten-free pasta during the *in-vitro* digestion process. Anti-nutritional factors in legumes, such as trypsin inhibitory activity and α-galactosides were reduced by up to 82% and 73%, respectively, by pasta processing and cooking. However, these processing steps had a minor effect on phytic acid. This study demonstrates the advantages of using legumes for the production of gluten-free pasta with a low glycemic index and high nutritional quality.

## Introduction

Wheat semolina has long been considered a central component of food, notably in pasta products. It contains gluten proteins, mainly composed of gliadins and glutenins (80%) that coagulate and form a strong viscoelastic protein network while the pasta is cooking, thereby trapping starch material inside. This unique feature of gluten proteins is responsible for the firm consistency and overall structure of pasta [1]. The particularly compact structure of wheat pasta is responsible for the slow digestibility of its starch content, hence promoting a low plasma glucose response resulting in a low glycemic index [2, 3]. However in some individuals, gluten ingestion causes a range of clinical disorders named “gluten-related-disorders” with a world prevalence of 5% [4]. These disorders including celiac disease, wheat allergy and non-celiac gluten sensitivity, manifest closed clinical symptoms. A restriction or total omission of gluten from the diet is the sole alternative [5, 6]. Thus, the demand and consumption of gluten-free food has increased [7]. However, in 2015, a study of 303 pasta products available in the Australian market revealed that commercial gluten-free pasta presented low nutritional properties, as they are generally poorer in protein (mean 6.1 g per 100 g) than classical no gluten-free pasta (mean of 12.6 g per 100 g) [8]. In addition, the removal of gluten from wheat flour was shown to increase the rate of amylolytic digestion *in-vitro*, and to enhance the glycemic response in bread *in-vivo* [9].

Legumes are gluten-free ingredients that could be used to produce gluten-free pasta of high nutritional quality. Legumes are rich in proteins (20–37%, w/w) [10] easily available [11], rich in dietary fibers (3–31% w/w) [12] and in resistant starch (RS, 11–20% w/w) [13]. The *in-vitro* and the *in-vivo* glycemic index can be reduced or maintained at its low value in legume enriched pasta [14–17] in comparison to their no-legume counterparts. In addition, legumes are also known to lower blood cholesterol and triglycerides [11]. Despite these interesting nutritional properties, raw legumes contain certain bioactive compounds considered as “anti-nutritional factors”. These compounds include protease inhibitors, phytic acid and α-galactosides, able to reduce protein digestibility, nutrient absorption and may be responsible for intestinal discomfort, respectively [18–20]. Despite these disadvantages, some of these anti-nutritional factors are now recognized to have beneficial effects on human health. Indeed, phytic acid has a preventive action against cancer by chelating metals involved in DNA damage [21]; whereas α-galactosides present some prebiotic activity [18]. The amount of bioactive compounds in legumes can be significantly reduced by food processing (soaking, fermentation and germination) and cooking [18, 20, 22–24]. However, some studies reported residual activity of trypsin inhibitors after cooking 20% chickpea enriched pasta [25]. In addition, even if richer in protein than cereals, legumes are mainly composed of soluble proteins (albumins and globulins). The incorporation of such soluble proteins in pasta weakens the protein network of legume enriched pasta, increases cooking loss and reduces resistance to breaking [26]. To the best of our knowledge, only one study has reported the use of legumes as the sole ingredient in gluten-free pasta [27], focused on the use of faba bean and on the impact of bioprocessing and fractionation of its grain on the textural and sensory properties of pasta and its *in-vitro* starch hydrolyses.

The objective of the present investigation was to explore and compare pasta made from three different types of legume, and particularly the link between their multiscale structure and their rheological, cooking, nutritional and anti-nutritional properties, notably the digestibility of their starch content and their anti-trypsic factors, phytic acid and α-galactoside contents. We also compared all three legume pasta with commercial gluten-free pasta made from cereals.

## Materials and Methods

Dehusked faba (F, *Vicia faba*) and organic green lentil (L, *Urvum lens L*.) flours were supplied by GEMEF (Aix-en-Provence, France) and Celnat Industries (Saint-Germain-Laprade, France), respectively. Black-gram (BG, *Vigna mungo*) grains from the market were dehusked and dry ground at Pondicherry University (Pondicherry, India). The particle size distribution (D50) of F, L and BG flours was 25, 22 and 50 μm respectively. The three flours originated from non-genetically-modified grains and were not subjected to any pretreatment. All legume flours were conserved at 4°C.

### Chemical and nutritional composition

Total starch content was determined with an enzymatic assay kit (Megazyme, Co. Wicklow, Ireland; AACC method 76–13.01). Individual neutral sugar composition was determined at INRA “BIA” (Nantes, France), after acid hydrolysis [28] of the samples, by gas chromatography of alditol acetates [29]. Uronic acid content was determined at INRA “BIA” (Nantes, France) in acid hydrolysis supernatant using an automated m-phenylphenol method and galacturonic acid as standard according to Thibault et al. [30]. Total protein content was determined using the Kjeldahl procedure (NF V 03–050, 1970) with a conversion factor of 6.25. Amino acid profiles were determined by Agrobio (Rennes, France) according to the CEE method 152/2009 (2009). Lipid content and fatty acid profiles were determined according to the French NF ISO 6492 and NF-EN-ISO 12966–2 norms (respectively) by Inovalys (Nantes, France). Total, soluble and insoluble fibers were determined by ISHA (Lonjumeau, France) according to the JORF (1986) method. All analyses were performed in duplicate on raw materials. Cooked (at their own optimum cooking time) pasta were subjected to starch, protein and amino acid analyses as described for raw materials. Total, soluble and insoluble non-starch polysaccharides were determined in duplicate by Englyst Ltd Carbohydrates according to Englyst et al. [31].

### Pasta production

One hundred percent legume flours (F, L or BG) were processed into spaghetti with a lab-scale pasta extruder (Sercom, Montpellier, France) as described in the FR 14 62811 patent, and dried (AFREM, Lyon, France) at 55°C for 12 hours to reach 11% moisture. All pasta was produced in triplicate, and the three batches of each kind of pasta were pooled into a single batch for further analysis. Pasta made from legume flours was compared to Schär (Burgstall, Italy) commercial (C) gluten-free spaghetti (8% protein, 78% starch and 2% fiber). The C gluten-free spaghetti was composed of a mixture of maize, millet, rice flours and cane sugar syrup. The diameter of the dried C pasta was 1.85 ± 0.03 mm and that of the legume pasta was 1.47 ± 0.03 mm.

### General appearance and color of the dried pasta

A picture of dried pasta was taken using a Samsung camera with a resolution of 16 megapixels. The color of dry spaghetti was determined with a Minolta Chroma Meter (Model CR-400, Minolta Co., Osaka, Japan) using the Hunter L*, a*, b*. L* values measure black to white (0–100); a* values measure redness when positive, and greenness when negative; b* values measure yellowness when positive, and blueness when negative. Four measurements were made of each kind of pasta (n = 4).

### Pasta cooking properties

The optimum cooking time (OCT) of each legume pasta was determined according to the AACC approved method (66–50). C pasta was cooked for 11 min, the OCT suggested by its manufacturer. Water uptake (% dry pasta) of each pasta was calculated as the difference in weight between cooked and dried pasta relative to the weight of dried pasta. Cooking losses (%, db) were calculated as the difference between the dry matter of each dried and cooked pasta, relative to the dry matter of dried pasta [26]. All the experiments were performed in triplicate.

### Protein size distribution in raw materials and in dried and cooked pasta

Two sequential protein extractions were performed in triplicate on raw material (F, L and BG flours), dry and freeze-dried cooked legume pasta. Proteins were first extracted in sodium dodecyl sulfate (SDS) to disrupt the electrostatic, hydrophobic and hydrophilic interactions between proteins. The second extraction was conducted in SDS/dithioerythritol (DTE) and then subjected to sonication to disrupt disulfide bonds. All protein extracts were analyzed by size-exclusion high performance liquid chromatography (SE-HPLC) according to the modified method of Morel et al. [32]. In our experiment, total protein extractability after the two extraction steps was found to be equal to the total protein content obtained with the Kjeldhal procedure in each of the analyzed fractions (flour, dried or cooked then freeze-dried pasta), which means that no remaining non-extractable proteins were found in all samples after the two extractions. Once corrected for the different solid-to-solvent ratios used during extraction, areas (in arbitrary units) of SDS-soluble and DTE-soluble proteins were expressed as the percentage of the corresponding total area calculated for F, L or BG flour proteins. SE-HPLC analyses of the commercial pasta were performed on dried and cooked then freeze-dried pasta, and the results are expressed as a percentage of total extractable proteins (in SDS/DTE) of dried pasta.

### Light microscopy of cooked pasta

Cooked (OCT) pasta sections (8 μm) were cut at -20°C using a microtome (Microm HM 520, Walldorf, Germany) with a cryoprotector (Cellpath, Newtown, UK). The sections were stained with 1 g/L fast green (Sigma Aldrich Co., USA) and 1:8 (v/v) diluted lugol (Fluka, Buchs, Switzerland) [16]. Bright field images were acquired using the multi-zoom AZ100M microscope (Nikon, Japan) equipped with a Nikon DSRi1 (Nikon, Japan) color digital camera. Observations were made with a plan fluor 5x objective and a fixed optical zoom of 8, resulting in a magnification of 40x.

### Determination of rheological properties of cooked pasta

Texture profile analysis (TPA) was performed, as described in Petitot et al. [26] on pasta cooked to OCT using a TA-XTplus (Stable Micro Systems, Scarsdale, USA) texture profile analyzer equipped with Texture Expert software (Stable Micro Systems, Scarsdale, USA). Pasta cohesiveness and springiness were then obtained [26]. The firmness of pasta was determined using the same texturometer, according to the AACC approved method (66–50), and is expressed as the maximum force (N) required to cut five strands of spaghetti positioned adjacent to another at a constant rate of deformation (0.17 mm/sec). Three replicates of two different cooking steps (n = 6) were performed for each experiment.

### Determination of anti-nutritional factors in raw materials and in cooked pasta

Trypsin inhibitor activity (TIA) was determined according to the standardized ISO 14902 method. Phytic acid was extracted and determined colorimetrically [33]. α-galactosides (raffinose, verbascose and stachyose) were extracted according to Hou et al. [34] and determined by Agrobio (Rennes, France) based on the AOAC 980.13 and AOAC 982.14 methods. All analyses were performed in duplicate on raw legume flours and on freeze-dried cooked pasta, except for TIA, which was performed in triplicate.

### *In-vitro* starch digestion of cooked pasta

*In-vitro* starch digestion was performed on pasta cooked to OCT by Englyst Carbohydrates LTD [35]. The glucose released from starch was measured by HPLC during timed incubation with digestive enzymes under standard conditions. The glucose released up to 20 min incubation corresponds to rapidly available glucose (RAG), and is expressed in g per 100 g available carbohydrates (total carbohydrates minus resistant starch). This fraction was found to be positively correlated with the postprandial glycemic index [35]. The glucose released from starch between 20 and 120 min incubation corresponds to slowly available glucose (SAG). The starch not digested after 120 min corresponds to resistant starch (RS).

### Statistical analysis

All data were subjected to analysis of variance followed by Fisher’s least significant difference (LSD) test to compare means at the 5% significance level using Statistica 8.0 software (Tulsa OK, USA). Correlation coefficients (r) are given.

## Results and Discussion

### Composition of legume flours and pasta

The composition of legume flours (faba bean, lentil and black-gram) used to produce pasta is presented in Table 1. Starch (46–58%, db), proteins (24–28%, db) and fibers (11–31%, db) were the main components of all legume flours. This composition differs from that of the wheat semolina usually used to produce pasta, which contains 13% protein, 78% starch and 2% fibers [26]. Among the legume flours, F contained the least protein (24%, db) but the most starch (58%, db). BG had the highest fiber content, the fibers being 87.5% insoluble the same as in F (89.7%) and L (88.5%) flours, and contained 2–3 times more soluble fibers than F and L flours. Considering the high galactose and arabinose measured, BG was probably made of mucilaginous polymers such as arabinogalactans, in accordance with reports in the literature [36]. The glucose measured in BG and F flours mainly originated from starch. In lentil flour, 16% of the total glucose did not originate from starch. It possibly originated from cellulose or hemicellulose material (such as xyloglucans, as higher xylose content was found in L than in F and BG flours). In L flour, uronic acid (composed of 75% galacturonic acid; result not shown) was also higher than in F and BG flours. It could originate from pectic substances [37]. Lentil grains are small and therefore have a high surface to volume ratio which increases the proportion of the seed coat to cotyledon thereby increasing the proportion of cell wall materials in the flour after milling. Regarding amino acid composition (Table 1), all the legume flours had higher essential amino acid contents than those recommended by the WHO/FAO/UNU for human adults [38], except for sulfur amino acids and valine in F proteins. Sulfur amino acid is known to be a limiting amino acid in legume proteins (*Lathyrus*, *Lens*, *Pisum* and *Faba* genera) [39]. However, in our study, proteins in L and notably in BG flours presented a complete essential amino acid profile compared to WHO/FAO/UNU recommendations. Amino acid composition of legumes can differ depending on the species and genotype of the legume and on environmental conditions. Amino acid composition can be improved using genetic engineering [40], but this was not the case in our study as we used non-genetically modified grains.

**Table 1:** Composition of legume flours.

		Legume flour		
Composition		F	L	BG
Starch (%, db)		57.6	48.6	45.7
Total fibers (%, db)		11.7	16.5	31.3
Insoluble fibers (% of total)		89.7	88.5	87.5
Soluble fibers (% of total)		10.3	11.5	12.5
Monosaccharides (%, db)				
Rhamnose		0.16	0.17	0.30
Fucose		0.00	0.06	0.05
Arabinose		2.36	2.38	5.06
Xylose		0.74	1.07	0.37
Mannose		0.38	0.29	0.54
Galactose		2.63	3.93	5.46
Glucose		57.54	57.69	47.49
Uronic acid		0.61	2.08	0.55
Proteins (%, db)		24.0	28.1	28.1
Essential amino acids (mg/g protein)	WHO/FAO/UNU* recommendation (mg/g protein)			
Histidine	15	34.6	26.4	28.9
Isoleucine	30	33.7	46.1	48.3
Leucine	59	71.1	78.1	85.3
Valine	39	37.1	52.6	56.1
Lysine	45	71.1	72.2	68.5
Cysteine + methionine	22	17.4	23.3	31.8
Tyrosine + Phenylalanine	30	74.0	79.0	86.9
Threonine	23	35.9	37.9	34.8
Tryptophan	6	10.8	8.9	12.2
Total lipids (%, db)		1.91	2.25	2.22
Fatty acids (% total lipid)				
Saturated fatty acids		15.0	13.4	20.1
Unsaturated fatty acids		85.1	86.7	79.9
Omega 3 fatty acids		3.0	10.9	50.4
Omega 6 fatty acids		51.9	46.9	11.4

All analyses were performed in duplicate.

*WHO/FAO/UNU (2007) pattern for adults [38]

F: faba, L: lentil and BG: black-gram.

Lipids in legume flour were low, ranging from 1.91 to 2.25% (db) mostly composed of unsaturated fatty acids that are susceptible to lipid peroxidation thus rendering several changes in lipids and other constituents (proteins, carbohydrates minerals and vitamins) possible during storage and processing [24]. The composition of unsaturated fatty acids differed depending on the legume flour considered. F flour and to a lesser extent L flour, were particularly rich in omega-6 fatty acids. Conversely, BG flour was rich in omega-3 fatty acids. A diet with a high ratio of omega-3 to omega-6 (higher than ¼) is associated with a reduced risk of many chronic diseases of high prevalence [41].

Concerning the composition of cooked pasta (Table 2), no significant change in the protein and starch contents was observed in comparison to legume flours except for a 3 g per 100 g (db) increase in the starch content of BG pasta (48.7% in BG cooked pasta vs. 45.7% in BG flour). This difference could be due to a higher loss of some soluble components other than starch during pasta cooking. Fibers could be affected. Total non-starch polysaccharide content was higher in BG than in F and L pasta, as already observed in the legume flours. In F and L pasta, the insoluble fraction of non-starch polysaccharides was only slightly modified (71.9% instead of 89.7% in F, and 82.6% instead of 88.5% in L) in the flour and in the cooked pasta. However, in BG pasta, the insoluble fraction only reached 39.7% versus 87.5% in the raw BG flour, showing that some insoluble non-starch polysaccharides solubilized during cooking. Overall, cooking legume pasta did not significantly reduce the amount of essential amino acids, which still conformed to WHO/FAO/UNU recommendations. Only sulfur amino acids in F cooked pasta were 19% lower than the recommended amount, as in legume F flour.

**Table 2:** Composition of legume and commercial gluten-free cooked pasta.

		Gluten-free pasta			
Composition		F	L	BG	C
Starch (%, db)		57.4	48.8	48.7	78.9
Total non-starch polysaccharides (%, db)		6.7	9.0	12.0	2.1
Insoluble (% of total)		71.9	82.6	39.7	52.1
Soluble (% of total)		28.1	17.4	60.3	47.9
Proteins (%, db)		23.4	27.6	28.3	8.2
Essential amino acids (mg/g protein)	WHO/FAO/UNU* recommendation (mg/g protein)				
Histidine	15	35.4	26.0	28.9	22.2
Isoleucine	30	37.9	48.8	48.1	39.2
Leucine	59	78.6	83.0	88.2	118.4
Valine	39	42.4	56.5	61.0	54.0
Lysine	45	68.7	68.3	71.3	20.3
Cysteine + methionine	22	17.8	24.0	30.9	64.6
Tyrosine + Phenylalanine	30	80.8	84.8	92.8	89.3
Threonine	23	38.8	38.8	34.9	31.6

All analyses were performed in duplicate.

*WHO/FAO/UNU (2007) recommendation for adults [38]

Tryptophan amino acid was not analyzed. F: faba, L: lentil, BG: black-gram and C: commercial pasta.

The commercial gluten free cooked pasta had 1.4 to 1.6 fold higher starch and 2.9 to 3.5 fold lower protein content than legume pasta. It also contained 3 to 6 fold less non-starch polysaccharides composed of 50% soluble and 50% insoluble fractions. The leucine content of C gluten-free pasta proteins was 1.5 fold higher than in legume pasta. Because the commercial gluten-free pasta was made exclusively from cereal, like most cereal proteins, it was richer in sulfur amino acid (2 to 4 fold more than legume pasta) but deficient in lysine content, therefore only covering 55% of the amount recommended by WHO/FAO/UNU).

### Appearance and color of pasta

**Fig 1:**
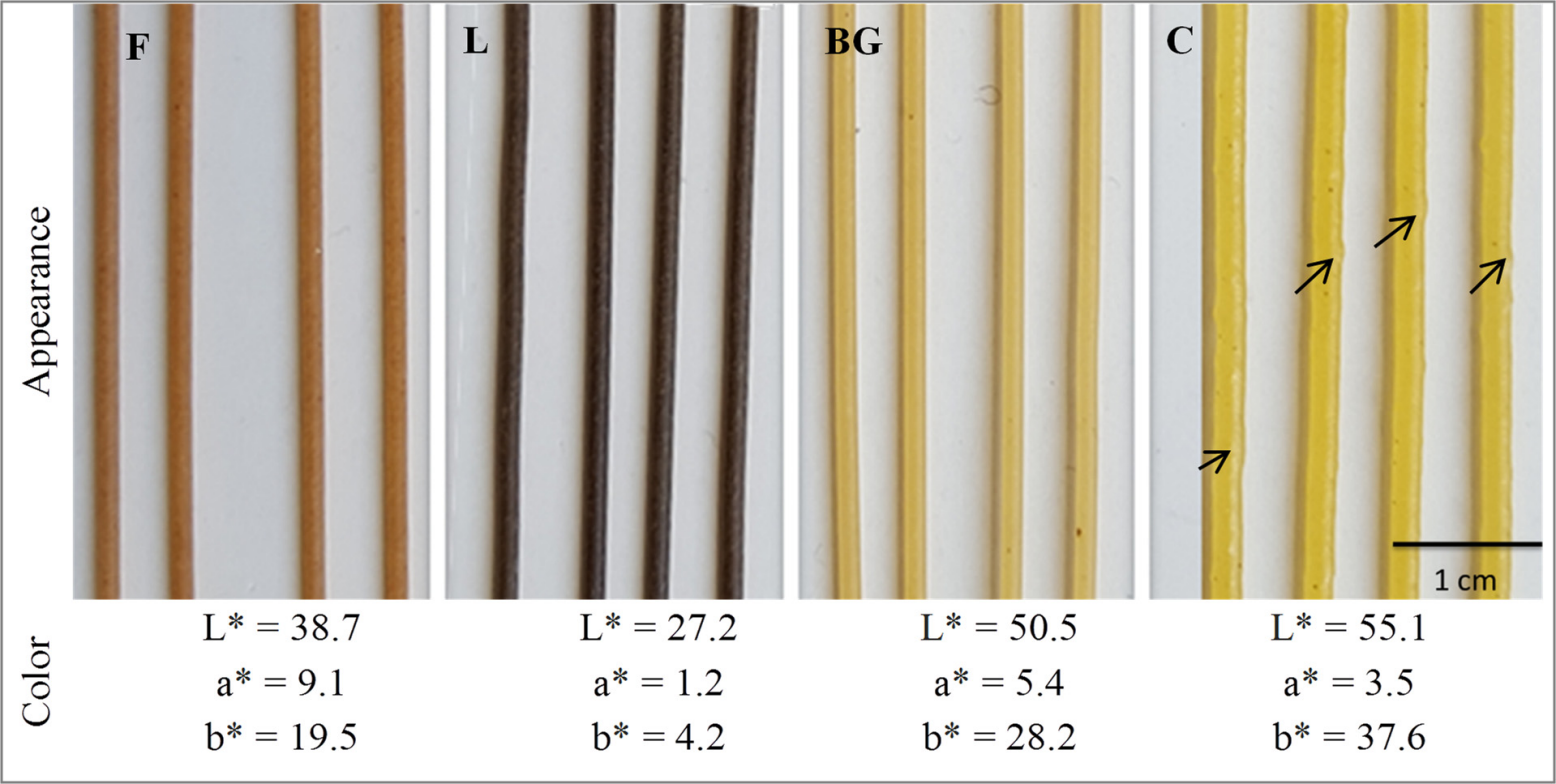
General appearance and color scores of dried pasta. F: faba, L: lentil, BG: black-gram and C: commercial pasta. Arrows denote bumps on the surface of C pasta. L* values measure black to white (0–100); a* values measure redness when positive, and greenness when negative; b* values measure yellowness when positive, and blueness when negative.

Fig 1 shows strands of dry pasta. All the legume pasta had a homogeneous appearance with a smooth surface. Their diameter was identical (1.47 ± 0.03 mm). Legume pasta differed in color. BG pasta had the highest brightness (L*) and yellowness (b*) followed by F pasta. L was the darkest pasta with the lowest yellowness and redness scores resulting in a green pasta. Redness (a*) was higher in F pasta followed by BG pasta. The color of pasta partially depends on the color of raw material used to produce the pasta [25]. Pasta color may also be related to enzymatic oxidation during mixing and the first steps of pasta drying [42] and to the vacuolar pigments (anthocyanin) present in legume flours [43]. C pasta had a rough surface and a larger diameter than legume pasta. Its color was characterized by higher L* and b* scores than all legume pasta with a lower a* score than BG and F pasta, resulting in a bright yellow pasta (Fig 1). Color scores of wheat pasta (results not shown) differed from all the gluten-free pasta with higher brightness (L* = 67.9) and lower redness (a = -0.3). The yellowness (b* = 25.1) was higher than F and L pasta, but lower than BG and notably C pasta. Even if the color of legume pasta is not the same as that of wheat pasta, their color is quite attractive as can be seen in Fig 1. Many different colored pasta are available on the market today (enriched in vegetable, fruits, ink, whole cereal, maize, etc.) which has contributed to consumer acceptance of diversified pasta products.

### Protein aggregation throughout the pasta process

**Fig 2:**
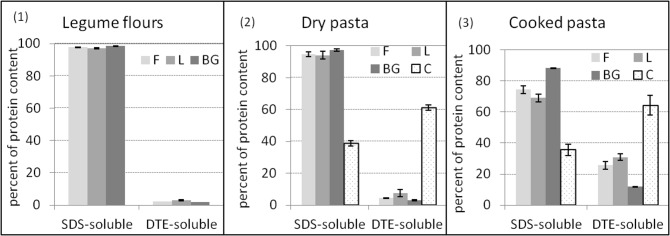
Changes in protein solubility in sodium dodecyl sulfate (SDS) and dithioerythritol (DTE). (1) Legume flours, (2) dry and (3) cooked pasta. F: faba, L: lentil and BG: black-gram flours. C: gluten-free commercial pasta. Results are means of three replicates, standard errors shown as vertical bars. Raw material and pasta were subjected to two sequential protein extractions first in SDS to disrupt the weak interactions, and then in SDS/DTE + sonication to disrupt disulfide bonds. All protein extracts were analyzed by SE-HPLC [32]. Areas of SDS-soluble and DTE-soluble proteins were expressed as the percentage of the total area corresponding to the total extractable proteins.

In order to determine the nature (strong or weak) of the protein network and the step at which it was built during the processing of gluten-free pasta, the degree of protein aggregation was investigated in all three legume (F, L and BG) pasta. It was determined on flours before processing and after drying and cooking of pasta, two steps known to determine the protein structure in wheat and wheat/legume mixed pasta [44, 45]. The gluten-free F, L and BG pasta were compared to gluten-free C (dry and cooked) pasta. The results obtained by SE-HPLC are presented in Fig 2. In all legume flours and pasta, the whole proteins were extracted in SDS and DTE with no non-extractable proteins recorded, meaning that the proteins were linked by electrostatic, hydrogen or disulfide bonds. Before processing (Fig 2.1), all legume flours (F, L and BG) showed a similar protein solubility profile; more than 97% being soluble in SDS with only 2–3% DTE-soluble proteins. Storage proteins in legumes are mainly composed of soluble globulins and albumin with minor glutelin and prolamine fractions [16], which could explain their high SDS solubility. After pasta processing and drying (Fig 2.2), only minor additional DTE-soluble proteins (+2.1, +4.5 and +1.1 additional percentage in F, L and BG, respectively) were formed at the expense of SDS-soluble proteins. Cooking the pasta (Fig 2.3) resulted in higher molecular changes in the protein solubility profiles of legume pasta than those observed during drying, especially in F and L pasta, where +21.2 and +23.4% (respectively) additional DTE-soluble proteins were formed at the expense of SDS-soluble protein. The same effect was observed but to a lesser extent in BG cooked pasta (+9% DTE-soluble proteins only). The low reactivity of BG pasta to binding through disulfide bonds was probably related to the smaller quantity of cysteine residue able to bind and to form cystine through the disulfide bond in BG protein than in F and L proteins (8.7, 9.6 and 11.2 mg/g; respectively; result not shown). In addition, the higher fiber content of BG (Table 1) could probably disrupt the protein network [16]. Whatever the legume pasta considered, the protein solubility profile differed significantly from that of regular pasta made from wheat semolina, where disulfide linked proteins represent 20% in raw semolina, 30% in dried pasta and 72 to 82% in cooked pasta [16, 46].

The results of protein aggregation in all gluten-free dried legume pasta demonstrated that cooking was the crucial step for the strengthening of the protein network. This result was different for C dried pasta based on cereals. The disulfide bonded protein (61%) pre-existed in dried C pasta (Fig 2.2), with no additional change observed during cooking (Fig 2.3). A stronger disulfide linked protein network governed the protein linkage in cooked C pasta and was 2.5, 2.1 and 5.5 fold higher than in F, L and BG pasta, respectively. This could be partly explained by the different nature of the proteins in legume (albumins and globulins) than in cereal (mainly glutelins and/or prolamins). Proteins in C pasta have higher sulfur amino acids content (64.6 mg/g protein) than in legume pasta (17–31 mg/g) (Table 2). A difference in drying temperature used for C pasta (not known) could also partly explain these results. Over a 90°C drying temperature, Petitot et al. [44] indeed reported similar 64% disulfide bonded protein in wheat pasta.

### Microscopic structure of pasta

**Fig 3:**
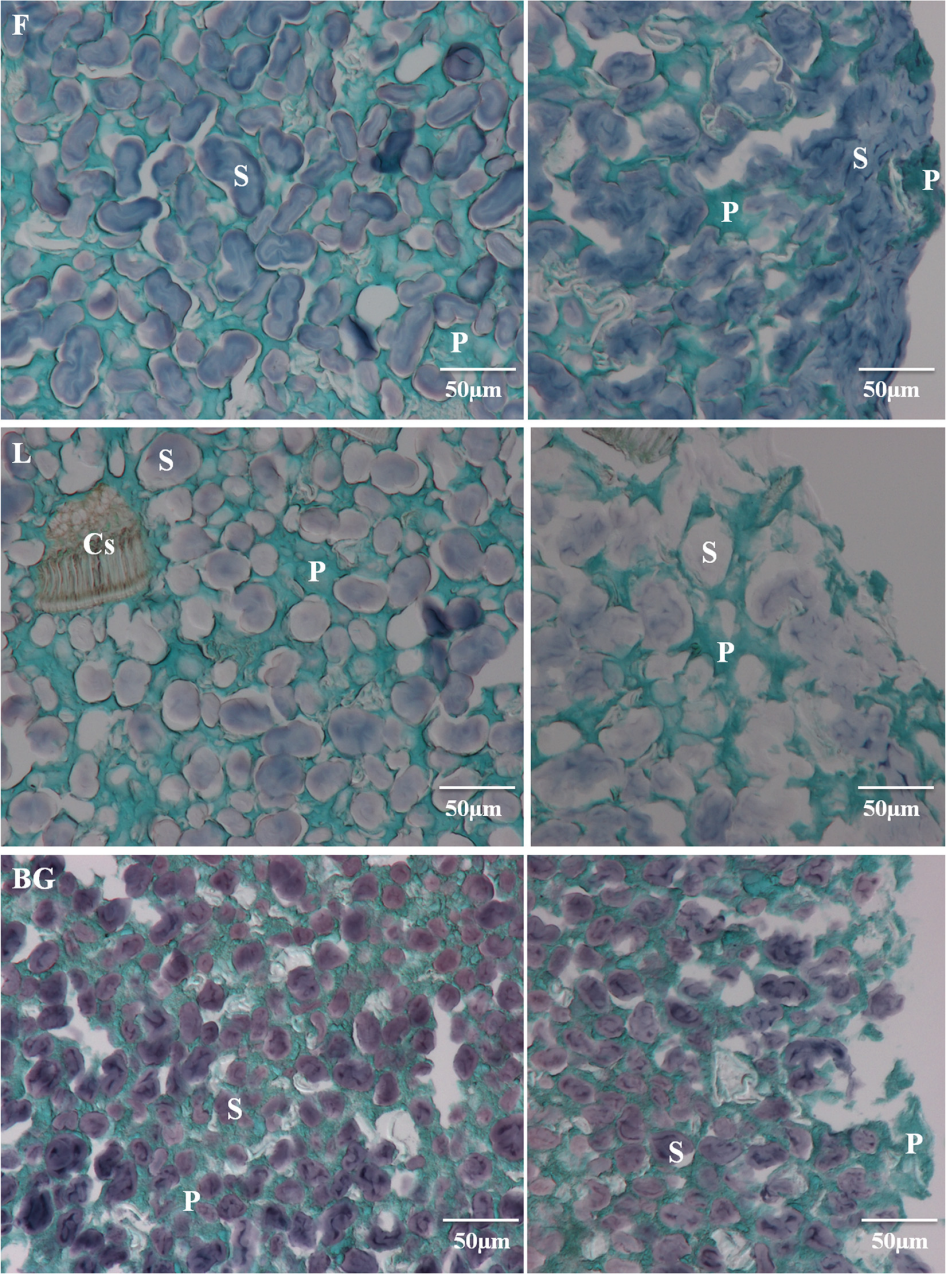
Light microscopy image of 100% legume cooked pasta. F: faba bean, L: lentil and BG: black-gram. The internal regions of the pasta are in the left panels and external regions in the right, panels. S, starch; P, protein and Cs, cell structure.

The microscopic structure of faba bean, lentil and black-gram legume cooked pasta is depicted in Fig 3. In all legume pasta, swollen starches (blue-violet) were included in a protein network (green), as reported by Rosa-Sibakov et al. [27] in 100% faba bean pasta. The protein (P) networks in all the legume pasta were more apparent and thicker than those reported in the literature for wheat pasta [16], in accordance with their higher protein content (24% for F and 28% for L and BG pasta versus 13% for wheat pasta). No distinction between F, L or BG pasta was found in the perceptible thicknesses of their protein network (P). The protein network of BG pasta appeared as small round structures as previously described in the literature [24]. The size and the shape of starch (S) differed depending on the raw material used to produce the pasta. BG presented smaller starch granules than F and L pasta. All the legume pasta had a high starch swelling rate even in the center of the pasta, with no major change in starch swelling from the center to the external region of the pasta strand. This result differs from results reported in the literature on wheat or wheat/legume mixed pasta, in which three main regions were distinguished from the outer to the central pasta strand according to the gradual starch swelling state [16, 46]. The lack of a strong covalently linked protein network in F, L and BG pasta, as revealed by SE-HPLC analysis, could have facilitated penetration of water to the core of the pasta and hence starch swelling. In the external region of F and L pasta, swollen starch granules met to form a continuous phase. Large cellular structures (Cs) were also observed in L pasta resulting from the cell wall, which is in accordance with the larger quantities of cellulosic and hemicellulosic materials in L in comparison to F and BG flours (Table 1).

### Cooking properties

Cooking properties (OCT, water uptake and cooking loss) of gluten-free legume pasta are listed in Table 3. OCT differed significantly according to the legume pasta considered. Two main groups were distinguished: the first one is formed by BG, which cooked in 3 min less than the second group formed by F and L pasta. The diameter of the pasta, which was identical in F, L and BG pasta, could not explain this huge difference. The more soluble and weakly linked protein network (Fig 2.3) and the higher fiber content, especially the water-soluble arabinogalactans (Table 1), of BG pasta in comparison to F and L pasta, could have facilitated penetration of the water to the core of the pasta. Rosa-Sibakov et al. [27] reported 9 min cooking time for 100% faba bean pasta with similar starch and protein composition (23.3 and 58.7%, respectively) to that of our F pasta. Around 10 min cooking time was reported for wheat pasta [27]. Pasta produced from 35% or 100% legumes (faba or split pea) reduced the pasta cooking time by 1 min in comparison to wheat pasta [26, 27]. The OCT of commercial pasta (11 min) was higher than the OCT of all legume pasta. This could be explained by two parameters: the bigger diameter and the higher starch content of C pasta (78%).

**Table 3:** Cooking and rheological properties of gluten-free legume and commercial cooked pasta.

		F	L	BG	C
Cooking properties	OCT (min)	9.5^a^	9.8^b^	6.6^c^	11*
	Water uptake (% dry pasta)	165^a^	154^b^	124^c^	103^d^
	Cooking loss (%, db)	14.35^a^	12.96^a^	10.35^b^	18.34^c^
Rheological properties	Firmness (N)	4.37^a^	5.92^b^	6.86^c^	10.13^d^
	Cohesiveness	0.70^b^	0.64^a^	0.64^a^	0.86^c^
	Springiness	0.75^a^	0.75^a^	0.85^b^	0.97^c^

Means with the different superscripts within a row are significantly different. F: faba, L: lentil, BG: black-gram and C: commercial pasta cooked to their optimal cooking time (OCT)

*OCT of C pasta was not subjected to variance analysis, as it was provided by the manufacturer.

Water uptake also differed significantly between F, L and BG pasta in the following order F > L > BG pasta (165, 154 and 124%, respectively). Water uptake of pasta is governed by different parameters including starch and fiber contents, the strength of protein network, OCT and the swelling power of its constituents. BG pasta had higher fiber, but lower starch content (Table 1) and a lower OCT, resulting in lower water absorption. L pasta had higher fiber but lower starch content than F pasta (Table 1), which could explain its lower water uptake than F pasta. C pasta absorbed less water (103%) than all the legume pasta during cooking. Its bigger diameter (1.85 mm) could delay the swelling of all its higher (78%) starch content. Indeed Laleg et al. [46] reported 186% water absorption for wheat pasta with the same (78%) starch content and a diameter of 1.56 mm when the pasta was cooked to its optimal cooking time + 1 min [46].

F and L pasta presented a statistically similar loss during cooking (Table 3). These values are higher than those reported in the literature for wheat pasta (5.6%) and for 35% or 100% legume (faba bean and split pea) pasta (7 to 12%) [26, 27], but lower than losses recorded for 15 to 30% common bean enriched wheat pasta (15–21% cooking loss) [47]. The lack of strong covalently linked protein network in our legume pasta, as demonstrated by SE-HPLC analysis, resulted in a weak overall pasta structure resulting in significant leaching out of particles during cooking. However, BG pasta, which had the most weakly linked protein network, also had the lowest cooking loss. This could be due to the lower OCT of BG pasta leading to less exposure of the surface of the pasta to the cooking water. In addition, this legume could contain a larger amount of arabinogalactan polymers (Table 1), which could play the role of stabilizer in pasta. The addition of 2% of hydrocolloids (pectin, guar or agar) to 15% chickpea pasta indeed reduced its cooking loss by slowing down the diffusion of the amylose molecules from the core to the surface of spaghetti [48].

All the legume flours presented a significantly lower cooking loss than C pasta. This could be due to the higher OCT of C pasta and to its lower protein content (8%) compared to legume pasta (24–28%). In addition, the tightly associated protein network pre-formed during the drying step (Fig 2.2) in C pasta could be less elastic and hence less deformable and breakdowns during starch swelling resulting in high cooking loss, as already reported in the literature [49]. Gluten-free pasta made from rice, from a mixture of rice and bean flour or from a mixture of chickpea unripe plantain and maize, lost 11–15% material [14, 50, 51].

### Rheological properties

Firmness (cutting force), cohesiveness (ability of the pasta to stick to itself) and springiness (ability of pasta to regain its original shape after compression) of legume (F, L and BG) and C cooked pasta are listed in Table 3. F, L and BG pasta presented a significantly different firmness with the highest firmness score for BG followed by L and F pasta. Pasta firmness was significantly (p<0.05) negatively correlated (r = 0.91) with water uptake. Pasta firmness is determined by the water taken up by the pasta and by the presence or the absence of a strong protein network. BG and L presented a similar cohesiveness, which was slightly lower than in F pasta. This lower cohesiveness could be mainly due to the presence of the cell wall structures observed by light microscopy in L pasta, and to the more weakly linked protein network in BG pasta (in comparison to L and F pasta). Like our results, Rosa-Sibakov et al. [27] reported a score of 0.74 cohesiveness for 100% F pasta, whereas higher cohesiveness (0.8) was reported for wheat pasta [44]. Springiness was higher in BG than in F and L pasta even though BG cooked pasta presented less disulfide linked proteins (Fig 2.3). This means that another type of interaction may be involved between the components of BG pasta. Indeed, arabinogalactan-rich carbohydrates, which were higher in BG than in F and L flours (Table 1), could form a network that improves the structure of the pasta, notably its elasticity, as reported in the literature on the use of hydrocolloids [48, 52].

All texture scores were significantly higher in C pasta than in all the legume pasta we tested. The lack of disulfide bonds (Fig 2.3) in F, L and BG pasta in comparison to C pasta explained the reduced springiness of the legume pasta. The lack of pasta elasticity is a major textural disadvantage associated with the depreciation of organoleptic properties in 30–35% legume (faba, pea, chickpea and lentil) enriched wheat pasta [25, 26]. The springiness of C pasta is in agreement with that of wheat pasta containing the same amount of disulfide bounds [44].

### Anti-nutritional factors of flours and pasta

Trypsin inhibitory activity (TIA), phytic acid and α-galactosides were determined in raw legume flours and in cooked pasta. The results are presented in Table 4. TIA ranged from 7.8 to 11.3 mg/g (db). BG flour contained 1.4 fold more TIA than F and L flours. TIA reported in the literature ranges from 2 to 18 mg/g for lentil [53–55], 15 mg/g for BG [54] and 3 mg/g for faba [56], depending on the type and variety of legume. Cooking pasta reduced TIA 3 fold in F and 5 fold in L and BG cooked pasta. The effectiveness of thermal treatment on TIA reduction is widely reported in the literature. Heat treatment (roasting, boiling or cooking) of legume seeds or flours reduced TIA by 38–96% [53–55]. Contrary to our results, Zhao et al. [25] reported no residual TIA in 15–20% spaghetti fortified with pea and lentil, because of the lower legume content and the higher drying temperature used, 70°C, versus 55°C in our study. The same author reported that pasta fortified with 20% chickpea retained 30% of its original TIA, in agreement with our results (18–32% residual TIA). The residual TIA in our pasta is in accordance with that reported by Frias et al. in 100% pea macaroni which retained 21 to 51% of the initial TIA activity after 30 min cooking [57]. Phytic acid contents (Table 4) of legume flours ranged from 14.5 to 18 mg/g in the order L > F > BG. Ranges of 8–12, 6–9 and 2–9 mg/g of phytic acid are reported in the literature [55, 58–60] for faba, lentil and black-gram seeds, respectively. The difference in legume cultivars, the climatic conditions and the application of fertilizers can affect the amount of phytic acid [24, 60]. Dried wheat and C pasta contained 6.5 and 7.25 mg/g (db) phytic acid, respectively (results not shown). Only a slight reduction in phytic acid content was observed in all cooked pasta in comparison to the corresponding flours. The pasta processing steps were less efficient in reducing phytic acid than fermentation and germination pre-processing [22, 61]. The use of low phytic acid mutant legumes [62] as done by Giuberti et al. [14] to produce gluten-free pasta, could be one way to prevent loss of nutritional quality, notably iron bioavailability, of pasta.

**Table 4:** Trypsin inhibitory activity (TIA), phytic acid and α-galactoside content of legume flours and cooked pasta.

Anti-nutritional factor (mg/g, db)		Legume flours	Cooked pasta
TIA* (n = 3)			
F		7.84^a^	2.48^a^
L		8.24^a^	1.52^b^
BG		11.26^b^	2.13^a^
Phytic acid (n = 2)			
F		15.31	14.91
L		17.97	14.76
BG		14.49	12.79
α-galactosides (n = 2)			
F	Raffinose	3.34	1.75
	Stachyose	8.75	4.86
	Verbascose	32.14	5.52
	Total	44.23	12.13
L	Raffinose	3.03	2.49
	Stachyose	37.86	10.88
	Verbascose	8.25	3.27
	Total	49.14	16.64
BG	Raffinose	1.25	1.10
	Stachyose	7.90	6.03
	Verbascose	47.50	26.50
	Total	56.66	33.63

*Means with the different superscripts within a column are significantly different

F: faba, L: lentil and BG: black-gram (BG)

Total α-galactoside ranged from 44 to 57 mg/g (db) in the order BG>L>F. Raffinose was a minor oligosaccharide in all legume flours. The concentration of stachyose and verbascose varied with the type of legume. Verbascose accounted for 73% and 84% of the total α-galactosides in F and BG flours, respectively, whereas the main oligosaccharide in L was stachyose with 77% of total α-galactoside. Our results concerning total α-galactoside contents are in agreement with those reported in the literature for faba (10 to 45 mg/g), lentil (18 to 75 mg/g) and for black-gram (33 to 81 mg/g) seeds [18, 58, 63, 64]. The percentage of each oligosaccharide is also reported to vary depending on the type of legume and agricultural factors such as irrigation conditions [63].

Pasta processing and cooking dramatically reduced the total amount of α-galactoside in F, L and BG by 73, 66 and 41%, respectively, which could be the result of heat hydrolysis or leaching in cooking water. This reduction was more efficient in L and F than in BG pasta probably because BG pasta was cooked for a shorter time (OCT of 6 vs. 9 min for the two others). The effect of cooking on the concentration of α-galactoside in legume seeds is well documented in the literature, and is reported to decrease α-galactoside considerably (from 25 to 100%), depending on the type of legume (faba, black-gram) and on the cooking conditions (20 to 60 min) [58, 64]. α-galactosides were not detected in cooked pasta supplemented with pigeon pea, because of the very low level of substitution (10%) and the fermentation preprocess applied to the legume seed prior to pasta manufacturing [22].

The effectiveness of pasta processing and cooking in our results also varied depending on the oligosaccharide considered, and was stronger in reducing verbascose in F (by 83%) and stachyose in L pasta (71%). This resulted in different oligosaccharide profiles in the cooked pasta than in the legume flours, notably in cooked F pasta, where the contribution of stachyose (40%, vs. 20% in legume flour) and Verbascose (46% vs. 73% in legume flour) to total α-galactosides was similar. Like in our study, verbascose oligosaccharide was reported to be subject of the largest reduction (36%) in soaked and cooked faba bean seed [58].

### *In-vitro* starch digestibility of cooked pasta

The results of *in-vitro* starch digestibility are presented in Table 5. A slight but significant difference was found in the amount of resistant starch (RS) between F, L and BG pasta (F>BG>L). RS was twice higher in all legume pasta than in C pasta. C pasta had a similar RS to RS values reported in the literature for wheat pasta [17]. A range of 0.88 to 1.69 g/100g (w.b) is reported in 35% faba bean and split pea enriched pasta [16, 17]. The amount of available carbohydrates was slightly but significantly higher in F (20.5%) than in L and BG pasta (19.1 and 18.9%, respectively). All legume pasta presented lower available carbohydrates than C pasta (34%), in accordance with their lower starch content (48–58% in legume pasta versus 78% in C pasta), and higher RS. F and L presented the lowest RAG (61.8 and 62.5%, respectively) and the highest SAG (36.4 and 36.0%, respectively). RAG values were lower than those reported in the literature for wheat pasta (67±0.7% of available carbohydrates) [17], but close to those reported for 35% faba enriched pasta (64.9±3.2% of available carbohydrates). BG pasta presented a significantly higher RAG and lower SAG than F and L pasta. This could be related to its lower aggregated protein network (SE-HPLC results) and smaller starch granules (microscope observation), two parameters that have been shown to increase starch digestibility [17, 65]. In addition, the black-gram starch has been reported to have a lower molecular weight of amylose and amylopectin than lentil starch, increasing its *in-vitro* glycemic index [65]. F and L pasta had lower RAG and higher SAG than C pasta. The higher protein content of legume flours could form a larger protein network in legume pasta, thereby slowing down starch hydrolysis [27]. SAG was lower in BG than in C pasta. C pasta presented similar *in-vitro* starch digestion parameters to those reported in the literature for wheat pasta [17]. The absence of gluten network did not increase the rate of hydrolysis in C pasta. In addition, even if legume pasta presented a weakly linked protein network, their RAG was either reduced (for F and L pasta) or closer to that of C pasta (for BG pasta). Legume starches are richer in amylose (30% to 37% [66]) than cereal starches (18% to 33% of amylose [67]). A higher amylose:amylopectin ratio means legume starch has a greater tendency to retrograde after the gelatinization-cooling steps, and to form a crystal structure that is resistant to digestive enzymes [24, 68]. In addition, the presence of anti-nutritional factors (such as phytic acid, which could inhibit α-amylase activity), which is particularly high in legume pasta, could affect the rate of starch digestion [65, 69, 70]. The lower RAG values obtained *in-vitro* for L and F pasta could be a predictor of a lower glycemic index.

**Table 5:** Resistant starch (RS), available carbohydrates, rapidly available glucose (RAG), and slowly available glucose (SAG) in cooked pasta.

Pasta	Dry matter (%)	RS (%, wb)	Available carbohydrates (%, w.b.)	RAG (% available carbohydrates)	SAG (% available carbohydrates)
F	31.93	1.16 ± 0.01^b^	20.50 ± 0.09^b^	61.83 ± 0.95^b^	36.43 ± 0.95^b^
L	36.15	0.99 ± 0.02^c^	19.14 ± 0.14^c^	62.53 ± 1.61^b^	35.99 ± 1.61^b^
BG	36.39	1.05 ± 0.03^d^	18.90 ± 0.15^c^	68.64 ± 2.26^a^	29.12 ± 2.26^c^
C	38.07	0.58 ± 0.02^a^	33.88 ± 0.66^a^	66.64 ± 1.34^a^	32.81 ± 1.34^a^

Means with the different superscripts within a column are significantly different. Analyses of starch digestibility were performed in triplicate. F: faba, L: lentil, BG: black-gram and C: commercial cooked pasta

## Conclusion

In this study, the structure and properties of gluten-free pasta made using 100% faba, lentil and black-gram flours were investigated. Whatever the legume used, the cooking and rheological properties of pasta, its microscopic structure and the strength of its protein network clearly differed from those of gluten-free cereal pasta. Legume pasta is up to 2 to 6 times richer in fiber and produced a weaker protein network, which was mainly formed during the cooking step in comparison to the cereal gluten-free pasta in which drying is the step that leads to the creation of the protein network. The texture of the legume pasta was therefore weaker with notably low springiness scores. Interestingly, all legume pasta, which is three times richer in protein than gluten-free cereal pasta, lost less material during cooking. If most legume pasta structure and properties were similar, some differences were noticeable depending on the legume used for pasta production. Black-gram pasta had better rheological and cooking properties related to its particular richness in arabinogalactan fibers.

From a nutritional point of view, in addition to their high protein content, the legume pasta also contained higher resistant starch than gluten-free cereal pasta. Furthermore, the remaining available starch in faba and lentil pasta was more slowly digested than the starch of gluten-free cereal pasta. This interesting *in-vitro* starch digestion profile could predict a lower glycemic index. However, even if legumes appear to be promising for the production of gluten-free pasta, their anti-nutritional factors need to be known. Trypsin inhibitory activity and the α-galactoside content can be drastically reduced during legume processing into pasta that includes a cooking step. Phytic acid was less affected by pasta processing, making germination, fermentation of legume grains or flours prior to pasta processing a promising option [22, 61].

In conclusion, the promising nutritional quality of legume pasta, notably their richness in protein and fibers and their potential low glycemic index could be of interest to expand the range of gluten-free cereal pasta available on the market today.
